# Assessment of *Mycobacterium bovis* Deleted in *p27-p55* Virulence Operon as Candidate Vaccine against Tuberculosis in Animal Models

**DOI:** 10.1155/2014/951978

**Published:** 2014-01-21

**Authors:** María V. Bianco, Simon Clark, Federico C. Blanco, Sergio Garbaccio, Elizabeth García, Angel A. Cataldi, Ann Williams, Fabiana Bigi

**Affiliations:** ^1^Instituto de Biotecnología, CICVyA-INTA, N. Repetto and De los Reseros, 1686 Hurlingham, Argentina; ^2^Public Health England, Porton Down, Salisbury SP4 0JG, UK; ^3^Instituto de Patobiología, CICVyA-INTA, N. Repetto and De los Reseros, 1686 Hurlingham, Argentina

## Abstract

A *Mycobacterium bovis* knockout in *p27-p55* operon was tested as an antituberculosis experimental vaccine in animal models. The mutant MbΔp27-p55 was significantly more attenuated in nude mice than its parental strain but more virulent than BCG Pasteur. Challenge experiments in mice and guinea pigs using *M. bovis* or *M. tuberculosis* strains showed similar protection conferred by MbΔp27-p55 mutant than BCG in terms of pathology and bacterial loads in spleen but lower protection than BCG in lungs. When tested in cattle, MbΔp27-p55 did not induce IL-2 expression and induced a very low production of IFN*γ*, suggesting that the lack of P27/P55 reduces the capacity of *M. bovis* of triggering an adequate Th1 response.

## 1. Introduction 

*Mycobacterium bovis* (Mb), the causative agent of bovine tuberculosis (BTB), infects cattle and other animals, including humans [1]. Although vaccination of cattle may represent an intervention strategy to reduce the impact of BTB on livestock productivity and human health in the developing countries, to date there is no available vaccine against BTB.

The gene that encodes P27/LprG constitutes a virulence operon together with *p55* that encodes an efflux pump or transporter [2]. Although P27 induces Th1 immune response, in BALB/c mice, when administrated as vaccine with BCG produced an adverse effect [2]. Moreover, coadministration of P27 with *M. tuberculosis* aggravates the infection [2], suggesting that this protein plays a role in *M. tuberculosis* infection by inducing increased suppression of the immune response. In this study we investigated the capacity of a *M*. *bovis* strain knockout in *p27*-*p55* operon to induce protective immune response in cattle and to vaccinate mice and guinea pigs against infection with virulent *M*. *bovis* and *M*. *tuberculosis*, respectively.

## 2. Results and Discussion

### 2.1. Examination of MbΔp27-p55 Virulence in Nude Mice

**Figure 1:**
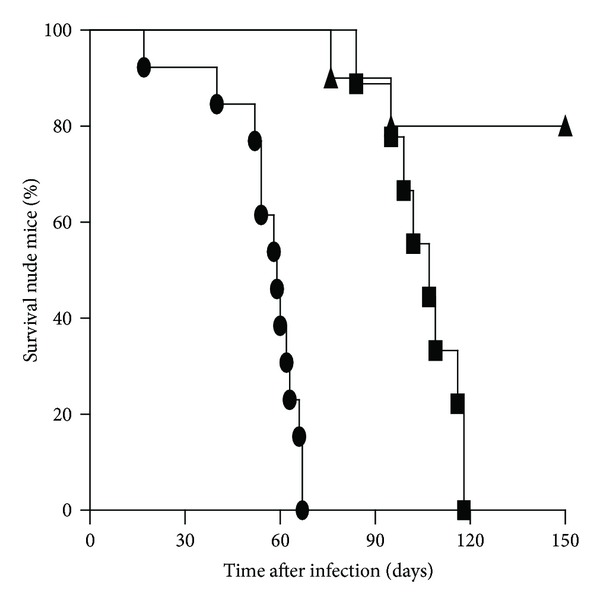
Survival of nude mice after intratracheal inoculation with 1.25 × 10^4^ CFU of WT (circles), MbΔp27-p55 (squares), or BCG (triangles) bacteria. Statistical analysis for survival curves was performed using Mantel-Cox tests (*P* < 0.001).

In order to comply with the safety requirements for a live TB candidate vaccine we evaluated the virulence of the MbΔp27-p55 in immunodeficient mice. Nude mice (10 per group) were infected with 12,500 colony forming units (CFUs) of the wild type or MbΔp27-p55 strains, and survival was assessed. The median survival of wild type-infected mice (59 days) was statistically different (*P* < 0.001) to that of MbΔp27-p55-infected animals (109 days) (Figure 1). This result demonstrates that MbΔp27-p55 is attenuated in the absence of a T-cell adaptive immune response and therefore is a safe candidate to be tested as a TB vaccine.

### 2.2. Evaluation of MbΔp27-p55 as TB Vaccine Candidate in Animal Models

**Figure 2:**
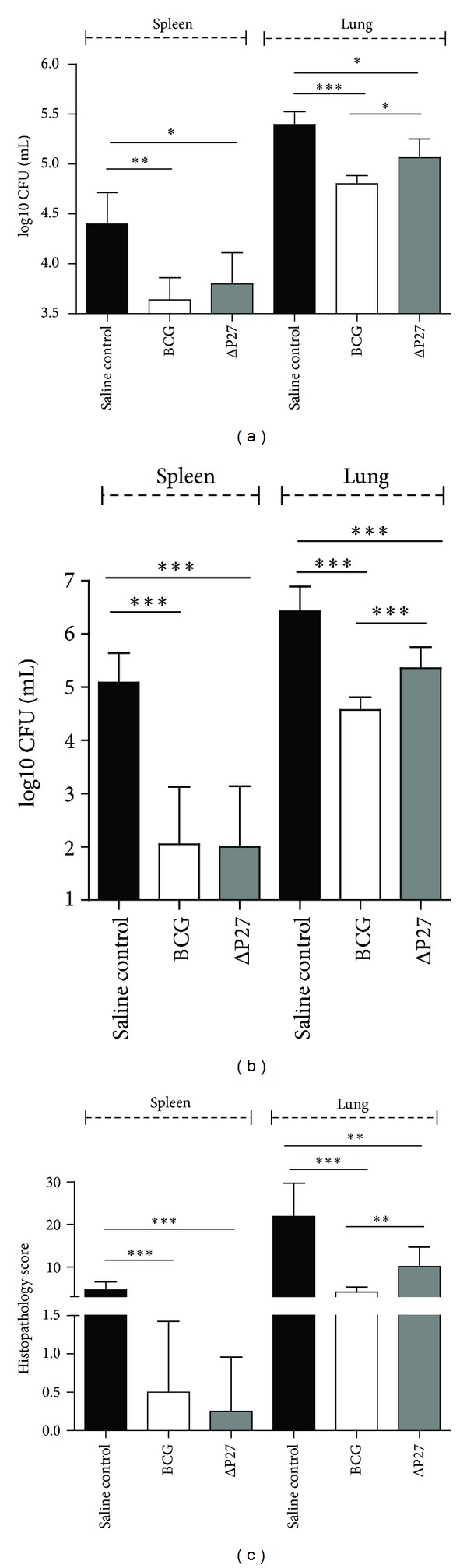
Protection assays in animal models. Organ bacillary loads after intratracheal challenge with *M*. *bovis* (a): BALB/c mice were vaccinated with the MbΔp27-p55 or BCG and compared with control nonvaccinated animals. CFUs were determined after 30 days of challenge. Organ bacillary loads (b) and histopathology (c) after aerosol challenge with *M*. *tuberculosis*: guinea pigs were vaccinated with the MbΔp27-p55 or BCG and compared with control nonvaccinated animals. Histopathology and CFUs were determined after 30 days of challenge. Data in (a) and (b) were analyzed using a two-tailed unpaired *t*-test, and data in (c) were analyzed using Mann-Whitney test (*P < 0.05, **P < 0.01, and ***P < 0.001).

The experimental challenge model of progressive pulmonary tuberculosis was used in this study [3]. Groups of BALB/c mice (7 per group) were vaccinated subcutaneously in the base of the tail with 100,000 bacilli of either the MbΔp27-p55 mutant or BCG Pasteur. At 60 days after-vaccination, all mice were challenged intratracheally with 125,000 CFUs of a virulent *M*. *bovis* strain. Mice were then killed at 30 days after-challenge. Levels of protection were determined by evaluating the numbers of viable *M*. *bovis* strain bacilli recovered from lungs and spleen. The numbers of CFUs cultured from the organs of each group are shown in Figure 2(a). Mice vaccinated with either BCG or MbΔp27-p55 were protected compared to saline control (*P* < 0.001), in both lungs and spleen. However, in the lungs, the protection conferred by the mutant was statistically lower than that of BCG (*P* < 0.05).

Groups of 8 Dunkin-Hartley guinea pigs were used to evaluate the efficacy of MbΔp27-p55 compared with BCG Danish 1331 both delivered subcutaneously in a single dose at a concentration of 5 × 10^4^ CFU. Twelve days after-immunization, animals were infected with a low aerosol dose of *M*. *tuberculosis* H37Rv. At 4 weeks after -challenge, guinea pigs were killed and organs were removed.

Protection was primarily assessed by measuring bacterial load in lungs and spleen and comparing the vaccinated groups of animals with the control group (saline). Guinea pigs vaccinated with either BCG or MbΔp27-p55 were protected compared to saline control (*P* < 0.001), in both lungs and spleen. However, in the lungs, the protection conferred by the mutant was statistically lower than that of BCG (Figure 2(b)).

A histopathological analysis of lungs and spleen lesions revealed that both vaccinated groups (MbΔp27-p55 and BCG) showed significantly reduced consolidation, foci of necrosis/caseation, and foci of calcification when compared with the unvaccinated group (Figure 2(c)). Again, guinea pigs vaccinated with BCG showed significantly reduced lung pathology when compared to animals vaccinated with the mutant strain.

### 2.3. Assessment of the Immune Responses Induced in Cattle after Inoculation of a *M*. *bovis* Strain Deleted in *p27* and *p55* Genes

In order to better understand the failure of MbΔp27-p55 to protect both mice and guinea pigs against tuberculosis, we used the cattle model to evaluate the immune response induced after infection with this mutant strain.

**Figure 3:**
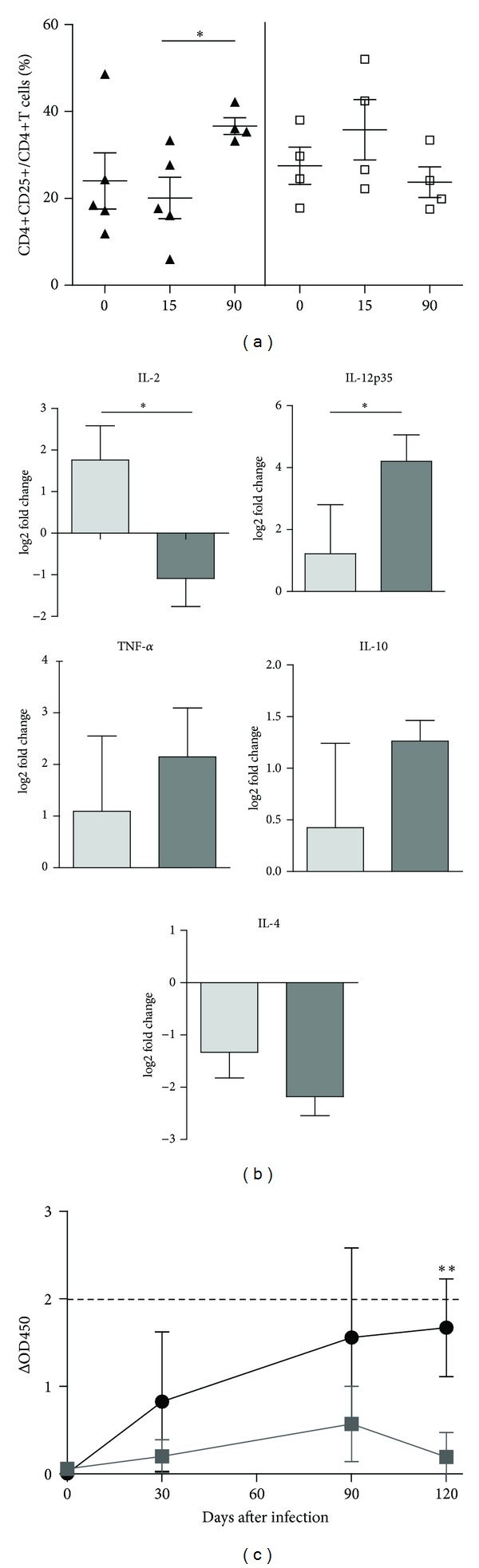
Response of *M*. *bovis*-infected cattle to PPDB. (a) Percentages of the activated lymphocyte cell subsets CD4+ of PBMCs stimulated with PPDB from animals inoculated with MbΔp27-p55 (*N* = 4, white square) or NCTC 10772 (*N* = 4-5, black triangle) at 0, 15, and 90 days after infection. Data were analyzed using the Wilcoxon matched pair test for cells with and without PPDB stimulation (*statistically significant *P* < 0.05). The means ± SEM are indicated. (b) Relative cytokine gene expression. Gene expression was measured in PBMCs from animals infected with either MbΔp27-p55 (*N* = 4, gray bars) or NCTC 10772 (*N* = 4, dark gray bars) stimulated with PPDB at 90 dpi. Relative gene expression was calculated using the 2-ΔΔCt method with E correction, using *pol II* and *gadph* mRNA expression as reference genes and the preimmune condition as the calibrator. Data were analyzed using a two-tailed unpaired Student's *t*-test (*P < 0.05). The bars indicate the average ratios of infected animals/uninfected animals ± SEM. (c) IFN-*γ* release in response to *M*. *bovis* antigens PPDB in blood from animals inoculated with MbΔp27-p55 (gray squares), WT (black circles) at different time points (0, 30, 90, and 120 dpi). Results are expressed as ΔO.D. (OD450 PPDB − OD450 PBS). Significance was determined by Mann-Whitney test (**statistically significant *P* < 0.01).

In peripheral blood monocyte cells (PBMCs) isolated 90 days after infection with the wild type strain, activation of CD4+ cells increased upon stimulation with PPDB (*P* < 0.01) (Figure 3(a)). In contrast, PBMCs isolated from animals infected with MbΔp27-p55 did not respond to specific stimulation with activation of CD4+ cells in any time point assayed.

We assessed the cytokine expression profile in PBMCs by measuring cytokine mRNAs after stimulating the cells with PPDB (Figure 3(b)). Values for sequential samples were normalized to values before inoculation for each animal. Given that there are available ELISA commercial assays to detect bovine IFN*γ* we used this methodology instead of quantification of IFN*γ* mRNA by RT-qPCR.

At 90 days after-infection (dpi), the expression of interleukin-2 (IL-2) in PBMCs was upregulated only in the group infected with the wild type strain, which is consistent with the CD4+ proliferative response, detected only in this animal group. Unexpectedly, only the group inoculated with the mutant responded to PPDB stimulation with production of IL-12 (*P* < 0.05), while the expression of TNF*α* was upregulated in both animal groups with no significant differences between them (Figure 3(b)).

The expression of IL-4, a Th2 cytokine, was downregulated in both groups (Figure 3(b)). This result is consistent with the low level of IFN*γ* detected at 90 dpi in both animal groups. It has been proposed that IL-4 is produced to compensate the inflammatory response induced by IFN*γ*.

At 120 dpi, the production of IFN*γ* in culture supernatant of PBMC stimulated with PPDB was significantly lower in the group inoculated with the MbΔp27-p55 mutant than in the group inoculated with the wild type strain. In fact, the group inoculated with the mutant strain produced very low quantities of IFN*γ* after PPDB stimulation throughout this study (Figure 3(c)).

Altogether, these results indicate that, although there is upregulation of IL-12 and TNF*α*, observed at 90 dpi, the lack of P27 and P25 in *M*. *bovis* reduces the capacity of the bacilli to induce a significant Th1 response when inoculated in cattle.

## 3. Conclusions

In this study we demonstrated that a *M*. *bovis* mutant in *p27-p55* operon did not confer better protection than BCG in both mice and guinea pigs. MbΔp27-p55 was more virulent than BCG in athymic mice, suggesting that its reduced protective capacity was not due to an inability to establish an infection. We found that the mutant induced in cattle the transcription of IL-12 and TNF*α*, two important Th1 cytokines. However, in contraposition, CD4+ cells from cattle inoculated with the mutant did not proliferate in response to specific stimuli, and the production of IFN*γ* in blood was nearly undetectable in this animal group. Therefore, altogether these results suggest that the lack of *p27-p55* operon reduces the capacity of *M*. *bovis* to induce an adequate Th1 response, underlining the immunogenic properties of P27. In the light of the results of this study, a *M*. *bovis* deleted in *p55* virulence gene carrying an intact *p27* gene would be an attractive candidate to be tested as TB vaccine.

## 4. Materials and Methods

### 4.1. Mouse Vaccination and Infections

Groups of female nude (N:NIH (S)-*FoxnI* ^nu^) mice of 6–8 weeks old were used to assess the virulence of MbΔp27-p55 strain.

BALB/c mice aging 6–8 weeks old were used for vaccination and challenge experiments. *M*. *bovis* NCTC 10772 strain (the parental strain of mutant MbΔp27-p55) was used as challenge strain. This experiment was repeated twice. Experiments with mice were performed in compliance with the regulations of Institutional Animal Care and Use Committee (CICUAE) of INTA. MbΔp27-p55 or BCG Pasteur were delivered subcutaneously in a single dose at a concentration of 1 × 10^5^ CFU. 60 days after vaccination the animals were infected with 1,25 × 10^5^ CFU of *M*. *bovis* by intratracheally instilation.

### 4.2. Guinea Pig Vaccination and Infection

Groups of 8 Dunkin-Hartley guinea pigs, weighing between 250 and 300 g (free of infection), obtained from a commercial supplier (Harlan, UK), were used to evaluate the efficacy of MbΔp27-p55 compared with BCG Danish 1331 (Statens Serum Institute, Copenhagen, Denmark), both delivered subcutaneously in a single dose at a concentration of 5 × 10^4^ CFU, and a negative control unvaccinated group. Guinea pig experimental work was conducted according to UK Home Office legislation for animal experimentation and was approved by the local ethics committee.

Animals were infected with a low aerosol dose (10–50 CFU retained dose in the lung) of *M*. *tuberculosis* H37Rv [4] 12 weeks after vaccination. Nose only aerosol challenge was performed using a fully contained Henderson apparatus as previously described [5, 6] in conjunction with the AeroMP (Biaera) control unit [7]. The aerosol was generated from a water suspension containing 5 × 10^6^ CFU/ml in order to obtain an estimated retained, inhaled dose of approximately 10–50 CFU/lung [7]. At 4 weeks after-challenge, guinea pigs were killed humanely by intraperitoneal injection of pentobarbital (Euthatal). Postmortem, lungs and spleens were taken and processed for bacteriology and histopathology analysis (subjective score), as described previously [8]. A significant reduction in CFU (bacterial load) and the nature and severity of the lesions (histopathology score) of vaccinated animals when compared with the control groups was considered a protective effect of the vaccine [9].

### 4.3. Cattle Infections and Immune Response Evaluations

Cattle infections were performed in compliance with the regulations of CICUAE and authorized by the National Service of Agricultural and Food Health and Quality (SENASA) and National Consultant Commission of Agricultural Biotechnology (CONABIA). Group of Holstein-Fresian calves (six months old) were inoculated intratracheally as described previously [10] with 10^4^–10^5^ CFUs of either *M*. *bovis* NCTC 10772 (*N* = 4-5) or MbΔp27-p55 (*N* = 4) and blood samples were taken at different points. After three months of infection, the calves were euthanized and then thin slices of lungs and lymph nodes of the head and pulmonary region were analyzed for granuloma formations. Only one of the animals inoculated with the wild type NCTC 10772 strain developed macroscopic lesions compatible with tuberculosis (data not shown). These lesions were located in retropharyngeal lymph nodes. No lesions were observed in animals inoculated with the mutant MbΔp27-p55. Flow cytometry determinations and cytokine expression analysis were performed as previously described [10]. IGRA Interferon Gamma (IFN-*γ*) release assays were performed on blood samples by using ELISA-based kit (Bovigam; Prionics) as previously described [10]. Duplicate samples for individual antigens were analyzed.
